# Amyloid-β peptide-induced extracellular S100A9 depletion is associated with decrease of antimicrobial peptide activity in human THP-1 monocytes

**DOI:** 10.1186/1742-2094-10-68

**Published:** 2013-05-30

**Authors:** Eun Ok Lee, Ji Hye Yang, Keun-A Chang, Yoo-Hun Suh, Young Hae Chong

**Affiliations:** 1 Department of Microbiology, School of Medicine, Ewha Medical Research Institute, Ewha Womans University, 911-1, Mok-6-dong, Yangcheonku, Seoul 158-710, Republic of Korea; 2 Department of Pharmacology, College of Medicine, Seoul National University, Seoul, Republic of Korea; 3 Department of Pharmacology, College of Medicine, Gachon University, Incheon, Republic of Korea

**Keywords:** Alzheimer’s disease, Aβ1-42, cytotoxicity, S100A9, Antimicrobial activity, Innate immune response

## Abstract

**Background:**

S100A9 protein (myeloid-related protein MRP14, also referred to as calgranulin B) is a reliable marker of inflammation, an important proinflammatory factor of innate immunity and acts as an additional antimicrobial peptide in the innate immune system. Evidence indicates that S100A9 contributes to Alzheimer’s disease (AD) pathology, although the precise mechanisms are not clear.

**Methods:**

We were interested to study the mechanisms of S100A9 release upon Aβ1-42 stimulation, the potential roles of extracellular S100A9 depletion in Aβ-induced cytotoxicity, and the interaction with innate immune response in THP-1 monocytic cells that have been challenged with mostly Aβ1-42 monomers instead of oligomers. We used protein preparation, Ca^2+^ influx fluorescence imaging, MTT assay, siRNA knockdown, colony forming units (CFUs) assay and western blotting techniques to perform our study.

**Results:**

Aβ1-42 monomers elicited a marked decrease of S100A9 release into the cell culture supernatant in a dose-dependent manner in human THP-1 monocytes. This reduction of S100A9 release was accompanied by an increase of intracellular Ca^2+^ level. Aβ1-42-mediated decrease of S100A9 release was not associated with Aβ1-42-induced cytotoxicity as measured by MTT reduction assay. This observation was confirmed with the recombinant S100A9, which had little effect on Aβ1-42-induced cytotoxicity. Moreover, depletion of S100A9 with siRNA did not significantly evoke the cell toxicity. On the other hand, Aβ1-42-induced extracellular S100A9 depletion resulted in decreased antimicrobial activity of the culture supernatant after Aβ1-42 stimulation. Immunodepletion of S100A9 with anti-S100A9 also decreased the antimicrobial peptide activity of the vehicle treated culture supernatant. Consistently, the recombinant S100A9 clearly elicited the antimicrobial peptide activity *in vitro,* confirming the observed antimicrobial activity of S100A9 in the culture supernatant.

**Conclusion:**

Collectively, our findings suggest that the mostly monomeric form of Aβ1-42 negatively regulates the innate immune system by down-regulating the secretion of S100A9, which is likely a main mediator of antimicrobial activity in the conditioned media of human THP-1 monocytes.

## Background

Alzheimer’s disease (AD) is the most common and still incurable form of dementia, which primarily affects the population over the age of 60 years. Amyloid beta (Aβ) deposition, neurofibrillary tangle formation and neuroinflammation are the major pathogenetic mechanisms that, in concert, lead to neocortical and hippocampal atrophy, memory dysfunction and decline of cognition in AD [1,2]. There are currently no curative or effective clinical treatments for AD [3].

The innate immune response and inflammatory signaling play determinant roles in brain homeostasis, neuroprotection and repair. However, altered or excessive signaling in these injury defense systems contributes to neuroinflammation and the irreversible degeneration of brain cells [4]. Extensive innate immune gene activation reflecting chronic innate immune activation could accompany brain aging, increasing vulnerability to cognitive decline and neurodegeneration, consistent with the emerging idea of a critical involvement of inflammation in the earliest stages of AD [5]. Thus, clinical pharmaceutical trials aimed at modulating the immune system in AD have largely focused on dampening down central proinflammatory innate immunity and the manipulation of systemic immunity, and its communication with the central nervous system (CNS) [6].

Calgranulins reflecting calcium-binding properties and high expression in granulocytes are comprised of three proteins: S100A8 (calgranulin A, also termed as MRP8), S100A9 (calgranulin B, also termed as MRP14) and S100A12 (Calgranulin C). They are predominantly expressed by neutrophils, monocytes and activated macrophages in inflamed tissue [7]. These S100 calcium-binding proteins are important molecular mediators in a range of diseases, including microbial infections. In particular, S100A9 protein is a reliable marker of inflammation and an important proinflammatory factor of innate immunity. Elevated plasma levels of S100A9 are associated with inflammatory disorders such as chronic bronchitis, cystic fibrosis and rheumatoid arthritis [8].

The extracellular roles of S100A9 in leukocyte migration and chemotaxis, leukocyte activation, oxidant scavenging, and their relevance in inflammatory processes are in particular implicated [7,9,10]. Recent reports have also suggested that S100A9 acts as an additional antimicrobial peptide in the innate immune system, which provides immediate protection for the host against microbial challenge by recognizing the presence of microorganisms and preventing their tissue invasion, thus limiting microbial proliferation and inflammation [11,12].

Altered expression/function of these S100 protein members [13] has been associated with neurological diseases such as cerebral ischemia [14] and traumatic brain injury [15]. Earlier studies demonstrated that S100 proteins assemble within neuritic plaques and reactive glia, which may serve to prolong neuroinflammation associated with the pathogenesis of AD [16,17]. Our recent study showed that S100A9 expression was increased in the brains of Tg2576 mice, as well as in AD brains, which proposed its potential role in the neuroinflammation related to the pathogenesis of AD [18,19]. Another recent study reported that S100A9 interacts with Aβ and induces fibrillization, further supporting its association with AD [20]. However, a mechanistic link between S100A9 and AD pathology, and the detailed molecular mechanism have not been clearly shown.

We focused our research on the mechanisms of S100A9 release upon stimulation with mostly Aβ1-42 monomers, the potential roles of extracellular S100A9 depletion in Aβ-induced cytotoxicity, and the interaction with innate immune response in THP-1 monocytic cells that have been challenged with Aβ1-42 monomers instead of oligomers. The results of the present study show that the mostly monomeric form of Aβ1-42 negatively regulates the innate immune system by down-regulating the release of S100A9, which is likely a main mediator for the antimicrobial action in the culture media of human THP-1 monocytes.

## Materials and methods

### Materials

Synthetic siRNA for S100A9 and the non-specific control pool were purchased from Santa Cruz Biotechnology (Santa Cruz, CA, USA). Lipofectamine 2000 was purchased from Invitrogen (Carlsbad, CA, USA). Anti-S100A9 was acquired from R&D Systems (Minneapolis, MN, USA). Horseradish peroxidase-conjugated anti-mouse IgG and anti-rabbit IgG were obtained from Jackson ImmunoResearch (West Grove, PA, USA). Actinomycin, inhibitor of *de novo* mRNA expression, and cycloheximide, inhibitor of protein synthesis, were obtained from Calbiochem (La Jolla, CA, USA). The 3(4,5-dimethylthiazol-2-yl)-2,5-diphenyltetrazolium bromide (MTT) was obtained from United States Biochemical (Cleveland, OH, USA). The Ca^2+^ ionophore, ionomycin, and an endoplasmic reticulum Ca^2+^ pump inhibitor, thapsigargin, were acquired from Sigma-Aldrich (St Louis, MO, USA). Anti-β-actin antibody and other chemicals, including 1,2-bis(o-aminophenoxy)ethane-N,N,N',N'-tetraacetic acid (BAPTA) and ethylene glycol tetraacetic acid (EGTA), were also acquired from Sigma-Aldrich.

### Preparation of Aβ peptides

Aβ1-42 peptide was purchased from American Peptide Company (Sunnyvale, CA, USA) and prepared before use as previously described [21]. Aβ1-42 peptide was dissolved at 5 mM in dimethyl sulfoxide and diluted at 250 μM in double-distilled water before experiments. This preparation contains the mostly monomeric form of Aβ1-42 and very small amounts of dimers with larger oligomers up to 6-mers [21].

### Preparation of recombinant S100A9 protein

Human recombinant (r) S100A9 was obtained from Dr Tessier at Laval University Hospital Center (Sainte-Foy, Québec, Canada), expressed in *Escherichia coli* and purified by previously defined protocols [22]. The purity of protein was verified by sodium dodecyl sulfate polyacrylamide gel electrophoresis (SDS-PAGE). Specificity of S100A9-mediated effect was controlled by THP-1 cell treatment with heat-inactivated rS100A9 (rS100A9_hi_) prepared by incubation at 85°C for 2 hours.

### Cell culture

The human monocytic cell line THP-1 was obtained from ATCC (Rockville, MD, USA) and maintained in RPMI-1640 containing 10% heat-inactivated fetal calf serum as previously described [21]. THP-1, a mononuclear cell line of human origin, has been widely used as a model of human monocytes/macrophages or microglia not only because of its functional and morphological similarities, including its capacity to activate signal transduction pathways, but also because of functional differences in the metabolism of rodent and human microglial cells as previously described [23].

### Experimental treatment

After being washed, THP-1 cells were incubated with serum-free RPMI-1640 supplemented with 0.5% glucose for 1 hour at 37°C before stimulation. The cells were then stimulated by the addition of the mostly monomeric form of Aβ1-42 peptide for 24 hours in the presence or absence of rS100A9 or rS100A9_hi_. In some experiments, cells were incubated with ionomycin or thapsigargin to determine the effect of increase of intracellular Ca^2+^ level. To deplete extracellular or intracellular Ca^2+^, cells were pretreated for 1 hour with ethylene glycol tetraacetic acid (EGTA) or BAPTA, and further incubated for 24 hours in the presence or absence of Aβ1-42 monomers. All concentrations were selected on the basis of the maximal effects of the drugs on their specified targets. Vehicles were treated identically, but did not contain Aβ1-42 or pharmacological agents as described above. Vehicle alone exerted no detectable effects on cell viability. After stimulation with Aβ1-42 and/or the specific agents for 24 hours, total cell lysate and the supernatant were prepared and stored at −20°C until use for quantification of S100A9 release by western blot analysis. The supernatant was also analyzed in parallel for antimicrobial activities.

### MTT assay

The viability of cells was analyzed by the MTT assay to assess mitochondrial dehydrogenase activity as previously described [24]. Only viable cells are able to reduce MTT into a formazan product by mitochondrial dehydrogenase. After 24 hours of treatment of THP-1 cells with Aβ1-42 and/or rS100A9, MTT was added to the medium (1 mg/ml) and incubated for 4 hours at 37°C. The medium was removed and the cells were diluted in 120 μl of 1 N HCl:isopropyl alcohol (1:24) and incubated for 30 minutes at room temperature with shaking. The relative formazan concentration of each supernatant was measured by determination of the absorbance at 570 nm in a microplate reader.

### Calcium imaging and fluorescence measurements

To visualize intracellular steady-state Ca^2+^ levels, THP-1 cells were stained by adding Fluo 3A in its acetoxymethyl ester form (Fluo-3 AM) to 5 μg/ml culture media throughout Aβ1-42 or vehicle treatment as previously described [18]. Ca^2+^ influx fluorescence images were captured after treatment as indicated. Images were recorded using an Axiovert 200 inverted microscope and analyzed with the KS 300 analysis program (Zeiss, Oberkochen, Germany). An increase in intracellular Ca^2+^ level in the different cultures was expressed as fold of the response of the vehicle treated controls for each individual experiment.

### siRNA studies

Synthetic siRNA for S100A9 and the non-specific control pool were purchased from Santa Cruz Biotechnology, and transfection of the RNA oligonucleotide was performed using Lipofectamine 2000. THP-1 cells were treated with Lipofectamine 2000 (mock transfection), siRNA or non-specific RNA pool at the concentrations indicated. After 24 hours of transfection, the cell viability was measured by the MTT method.

### *E coli* culture and treatment

*E coli* strain LE392 was used throughout this study. Colonies from agar were transferred by sterile loop to growth media and incubated aerobically in Luria Broth (Conda, Madrid, Spain) for 2 hours at 37°C, to generate mid-logarithmic growth cultures for use as inoculates in experiments. Bacteria inoculum cell densities were normalized to 5 × 10^5^ cells/ml immediately before use. After stimulation with Aβ1-42, the supernatant collected was mixed with *E coli* in the ratio of 1:1. rS100A9 and rS100A9_hi_ were directly diluted into serum-free RPMI-1640 and also mixed with *E coli*. All stocks were incubated for 2 hours at 37°C. In some experiments, rS100A9 was preincubated with anti-S100A9 antibodies for 2 hours at 37°C before use. Experiments included control serial dilutions of medium or buffer vehicle alone.

### Colony forming unit (CFU) assay

Serial dilutions of incubants were prepared and streaked onto the surface of Luria broth agar (Miller’s LB agar). The agar plates were then incubated overnight at 37°C and CFUs counted as previously described [25].

### Preparation of human peripheral blood mononuclear cells (PBMC)

Human PBMC were isolated from peripheral blood of healthy subjects as previously described [24] and used as a positive control for S100A9 in western blot analysis. Preparations contained approximately 10% monocytes, 90% lymphocytes and <1.5% granulocytes.

### Electrophoresis and western blotting

Immunoblotting was conducted as previously described [24,26]. Equal quantities of sample proteins were separated on the basis of molecular weight by 10% SDS-PAGE and transferred to polyvinylidene difluoride membranes (Millipore, Bedford, MA, USA), which were subsequently blocked for 0.5 hours with 3% milk in Tris-buffered saline with Tween 20. The membranes were then probed with primary antibody diluted with 1% milk and incubated overnight at 4°C. Signals were acquired with an enhanced chemiluminescence system after incubation with horseradish peroxidase-conjugated secondary antibodies (Jackson ImmunoResearch). Densitometric values were normalized versus β-actin.

### Statistical analyses

Differences between groups were evaluated for statistical significance using one-way analysis of variance (ANOVA) with a Student’s t-test. Null hypotheses of no difference were rejected if *P* values were less than 0.05.

## Results

### Aβ1-42 reduced extracellular release of S100A9 in human THP-1 monocytes

**Figure 1:**
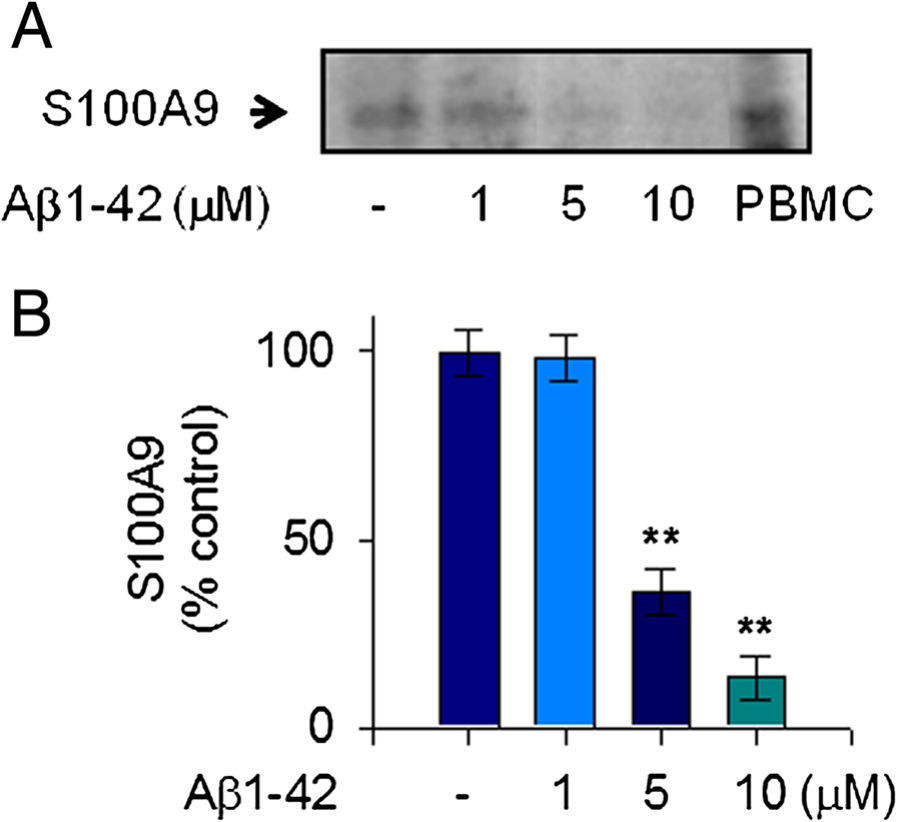
**S100A9 release in response to Aβ1-42 monomers in human monocytic THP-1 cells.** To measure the extracellular release of S100A9 in response to mostly monomeric Aβ1-42 stimulation, THP-1 cells were incubated with either vehicle only (−) or increasing amounts of Aβ1-42 for 24 hours in serum-free RPMI-1640 medium supplemented with glucose (0.5%). (**A**) The cell-free conditioned media were examined for S100A9 via protein immunoblot. Positive control for S100A9 was shown in human PBMC whole cell lysate. Aβ1-42 decreased S100A9 release in conditioned media in a dose-dependent manner. Results are representative of three independent experiments. (**B**) Densitometric quantification of analyses of (**A**), showing the levels of S100A9 release. All data are presented as the means ± SEM (n = 3). **P <0.01, versus vehicle treated samples. PBMC, peripheral blood mononuclear cells.

**Figure 2:**
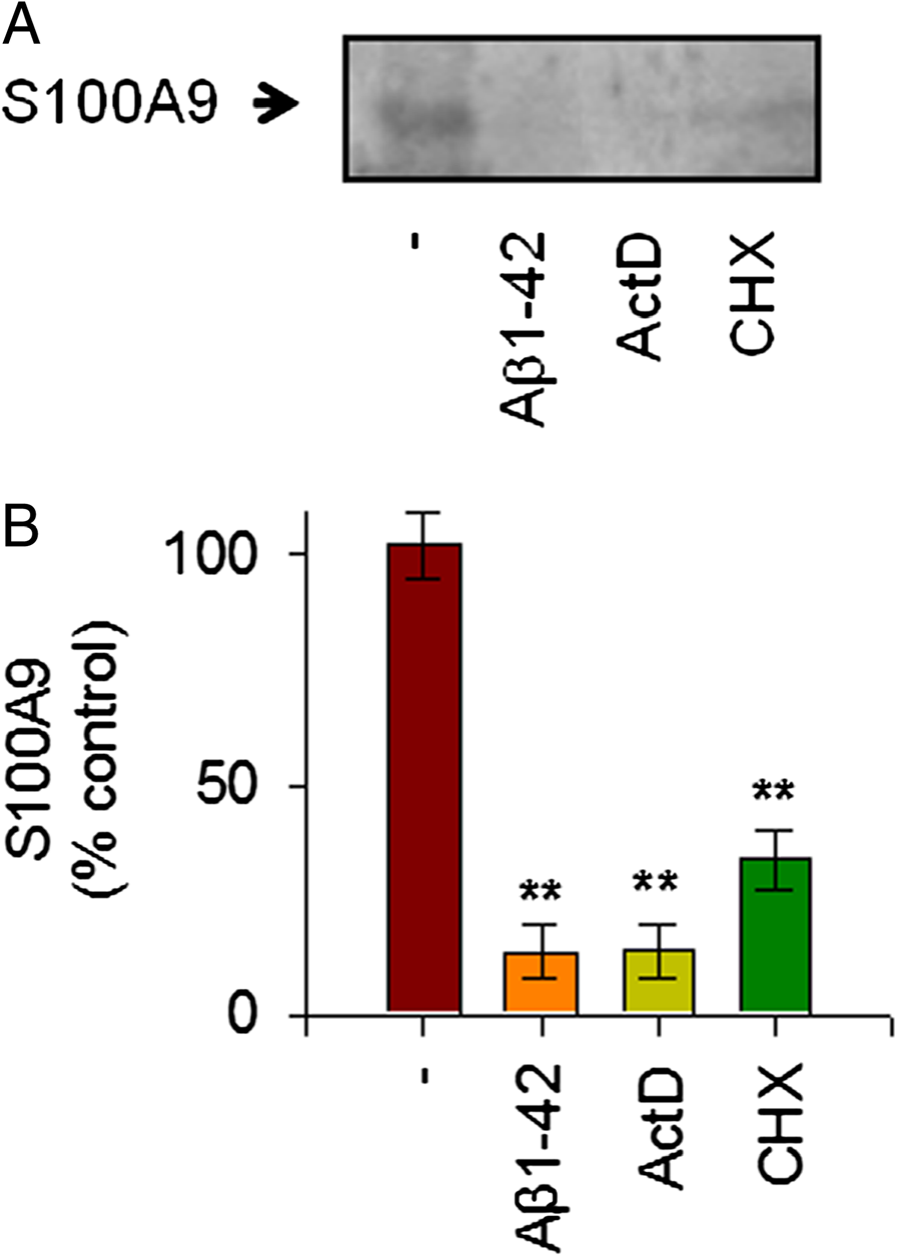
**S100A9 release process was dependent on transcription and translation processes in human monocytic THP-1 cells.** THP-1 cells were incubated with Aβ1-42 (10 μM), actinomycin (ActD, 100 nM) or cycloheximide (CHX, 1 μM) for 24 hours. (**A**) Western blot analyses were conducted to determine the effects of actinomycin or cycloheximide on S100A9 release in the conditioned media, as described in Figure 1. S100A9 secretion was reduced when *de novo* mRNA expression and protein synthesis were inhibited. Representative gels from three experiments are shown. (**B**) Quantitative analysis of (A), showing the levels of S100A9 release. All data are presented as the means ± SEM (n = 3). **P <0.01, versus vehicle treated samples. ActD, actinomycin; CHX, cycloheximide.

To clarify the pathological mechanism related to S100A9 in AD, we measured the extracellular release of S100A9 in response to stimulation with Aβ1-42 in human THP-1 monocytes. We used Aβ1-42 monomers instead of oligomers. The treatment of THP-1 cells with Aβ1-42 monomers significantly reduced the release of S100A9 at 24 hours in the conditioned media of THP-1 cells. This Aβ1-42-mediated decrease of S100A9 secretion occurred in a dose-dependent manner and maximal reduction of S100A9 secretion was found to occur at a concentration of 10 μM Aβ1-42 (Figure 1A, B). Notably, S100A9 secretion was consistently reduced when *de novo* mRNA expression and protein synthesis were inhibited by actinomycin D and cycloheximide, respectively. Thus, our data confirmed that Aβ1-42 elicited a marked decrease of the extracellular S100A9 release in a dose-dependent manner in human THP-1 monocytes, and that reduction of S100A9 release is dependent on both transcriptional and translational activities (Figure 2A,B).

### Intracellular Ca^2+^ level is involved in Aβ1-42-induced depletion of extracellular S100A9

**Figure 3:**
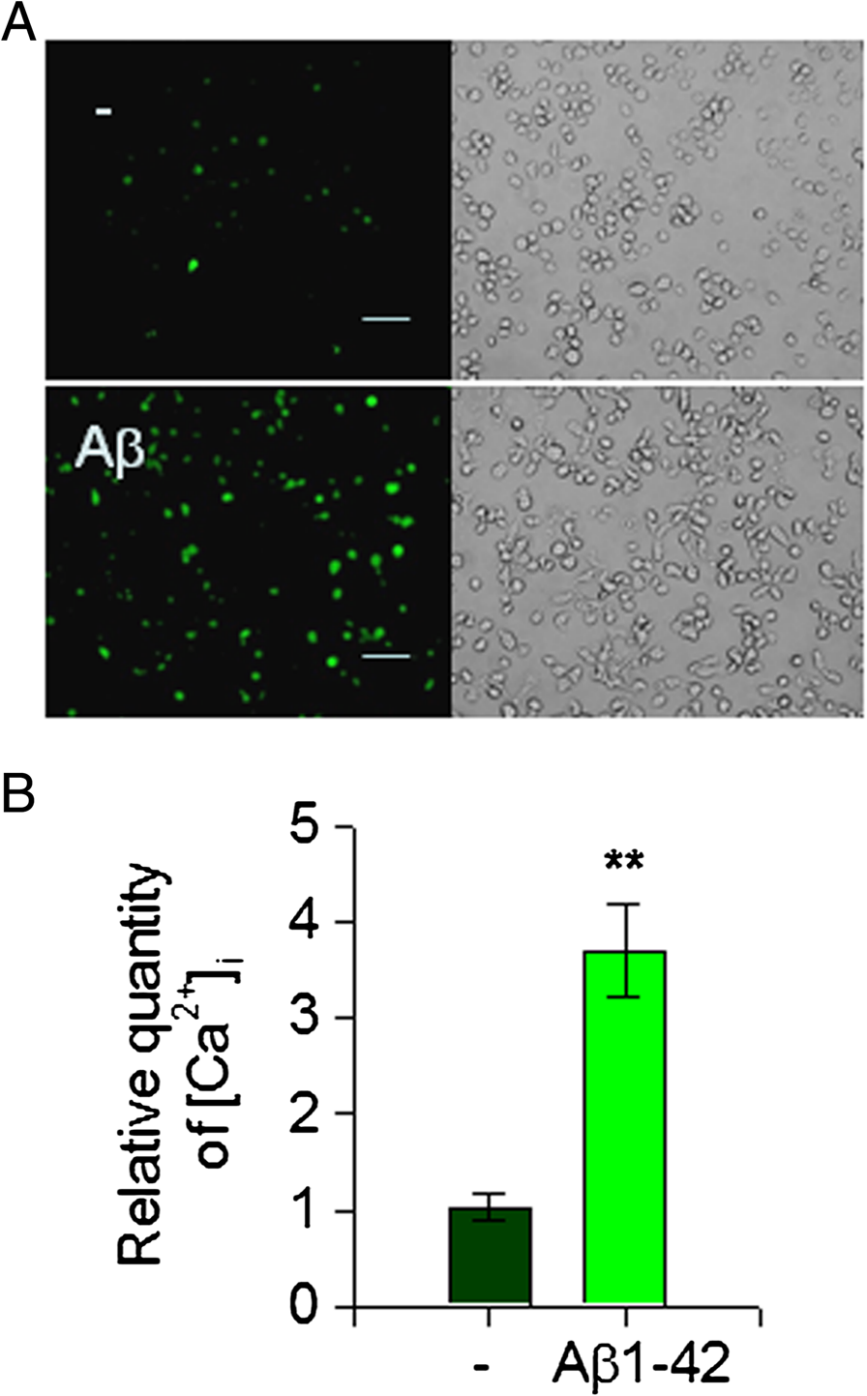
**Aβ1-42 monomers increased [Ca**^**2+**^**]**_**i **_**in human monocytic THP-1 cells.** (**A**) [Ca2+]_i_ images obtained by Fluo-3 AM at 24 hours after treatment with either vehicle only (−) or 10 μM Aβ1-42 monomers (Aβ) in THP-1 cells. Scale bars represent 50 μm. (**B**) The histogram showing the ratio of [Ca2+]_i_ levels to vehicle treated group. All data are presented as the means ± SEM (n = 3). **P <0.01, versus vehicle treated samples. Aβ, amyloid beta.

**Figure 4:**
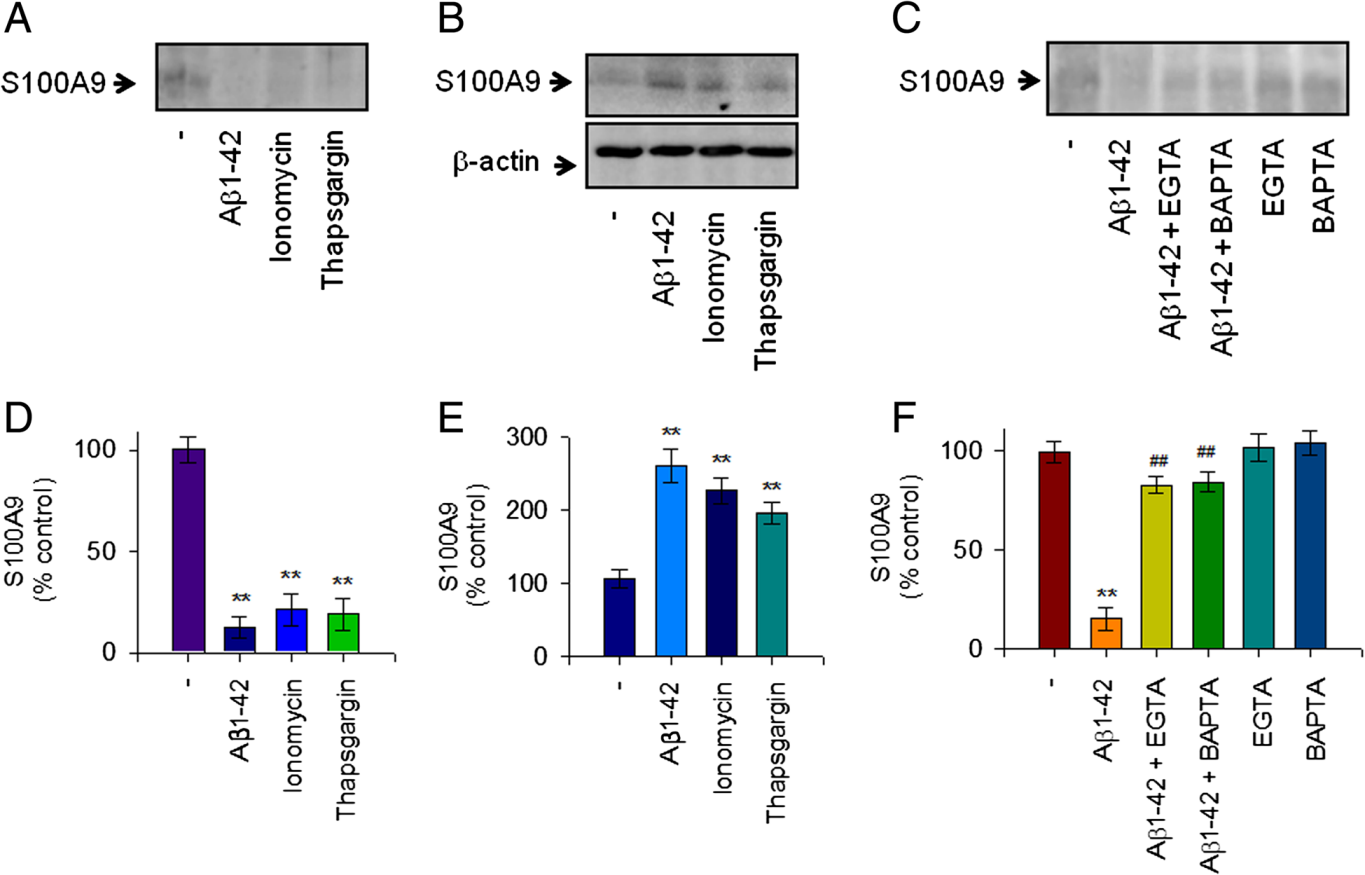
**The decreased extracellular S1009A in response to Aβ1-42 monomers is dependent on increase of intracellular Ca**^**2+ **^**level in human THP-1 monocytes.** THP-1 cells were incubated with Aβ1-42 (10 μM), actinomycin (ActD, 100 nM) or cycloheximide (CHX, 1 μM) for 24 hours. Western blot analyses were conducted to determine the effects of actinomycin or cycloheximide on S100A9 release in the conditioned media, as described in Figure 1. Ionomycin and thapsigargin mimicked the Aβ1-42-evoked response. THP-1 cells were incubated with Aβ1-42 (10 μM), ionomycin (2 μM) or thapsigargin (2 μM) for 24 hours. (**A**) The conditioned media and (**B**) total cell lysate were examined for S100A9 by western blot analyses. (**C**) THP-1 cells were pretreated with EGTA (0.5 mM) or BAPTA (10 μM) for 1 hour followed by incubation with either the vehicle only (−) or Aβ1-42 (10 μM) for 24 hours. Western blot analyses were conducted to determine the role of intracellular Ca^2+^ levels in Aβ1-42-mediated reduction of S100A9 release. Ionomycin and thapsigargin, which elevate intracellular Ca^2+^ level, mimicked the Aβ1-42-evoked response, decreasing S100A9 secretion. In contrast, the Aβ1-42-mediated decrease of S100A9 secretion was attenuated by either depletion of extracellular Ca^2+^ with EGTA or chelation of intracellular Ca^2+^ by BAPTA. Representative gels from three experiments are shown. (**D**, **E**, **F**) Quantitative analysis of (A, B, C) showing the levels of S100A9 release. All data are presented as the means ± SEM (n = 3). **P <0.01, versus vehicle treated samples; ^##^*P*<0.01, versus Aβ1-42 treated samples. ActD, actinomycin; BAPTA, 1,2-bis(o-aminophenoxy)ethane-N,N,N',N'-tetraacetic acid; CHX, cycloheximide; EGTA, ethylene glycol tetraacetic acid.

The increase of [Ca^2+^]_i_ may initiate the inflammatory response in activated microglia [27]. We observed that 10 μM Aβ oligomers extensively increased the level of [Ca^2+^]_i_ in murine microglial BV2 cells as evaluated using the Fluo-3 AM method [18]. Thus, we investigated the role of intracellular Ca^2+^ levels in Aβ1-42-mediated reduction of S100A9 release and found that intracellular Ca^2+^ level is involved in human monocytic cells. We also observed that 10 μM Aβ1-42 monomers significantly increased intracellular Ca^2+^ levels in THP-1 cells as measured by Fluo-3 AM (Figure 3A,B). Furthermore, treatment of THP-1 cells with the Ca^2+^ ionophore, ionomycin, which induces [Ca^2+^]_i_ elevating intracellular Ca^2+^ concentration, induced the Aβ1-42-evoked response decreasing the release of S100A9 (Figure 4A,D). Moreover, thapsigargin, an endoplasmic reticulum Ca^2+^ pump inhibitor, which induces an increase of intracellular Ca^2+^ level, also mimicked the Aβ1-42-evoked effects. Concomitantly, the intracellular levels of S100A9 were increased in THP-1 cells treated with either ionomycin or thapsigargin as observed in Aβ1-42 treated cells (Figure 4B,E). However, the Aβ1-42-evoked response was significantly attenuated by either depletion of extracellular Ca^2+^ with EGTA or chelation of intracellular Ca^2+^ by BAPTA (Figure 4C,F). Together, these findings suggest that extracellular depletion of S100A9 in response to Aβ1-42 monomers is dependent on an increase of intracellular Ca^2+^ and S100A9 levels in human THP-1 monocytes.

### Aβ1-42-induced depletion of extracellular S100A9 was not associated with Aβ1-42-dependent cytotoxicity

**Figure 5:**
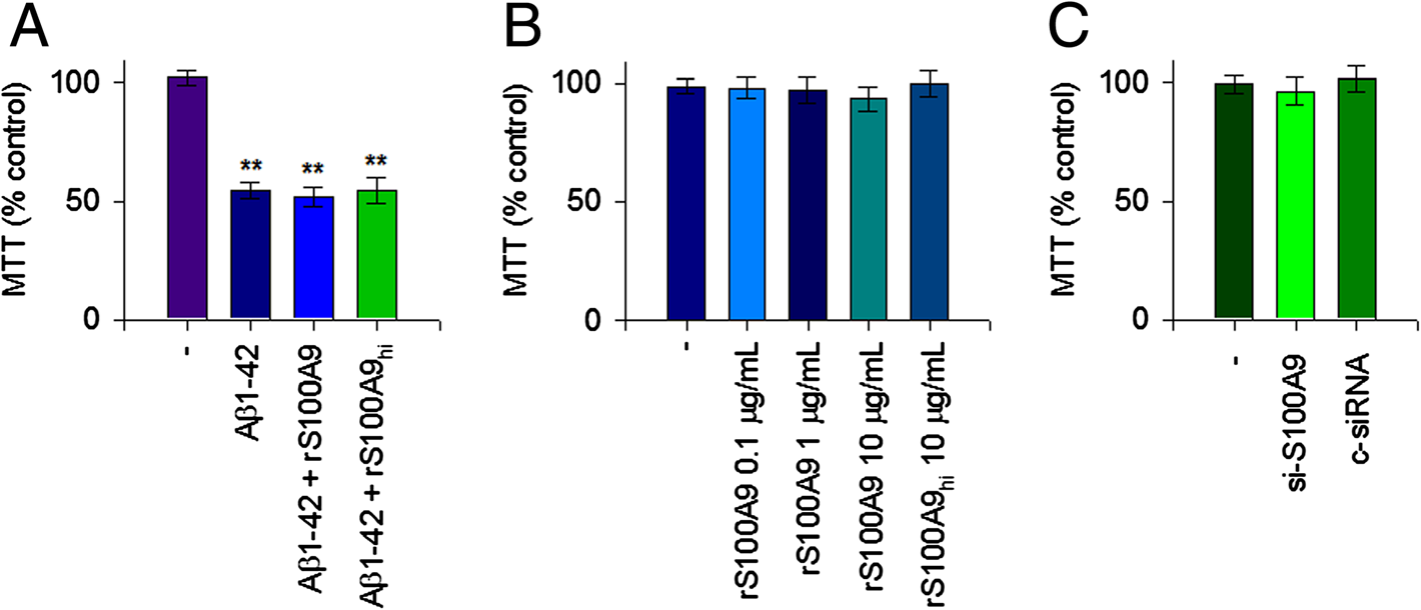
**Extracellular S100A9 depletion by Aβ1-42 monomers was not associated with Aβ1-42-induced cytotoxicity.** (**A**) To investigate the role of extracellular S100A9 depletion related to the Aβ1-42-induced cytotoxicity, THP-1 cells were incubated for 24 hours with Aβ1-42 (10 μM) in the presence of 10 μg/ml recombinant S100A9 (rS100A9) or heat inactivated rS100A9 (rS100A9_hi_). (**B**) THP-1 cells were also incubated with increasing amounts of rS100A9 alone as indicated for 24 hours. (**C**) THP-1 cells were transfected with S100A9 siRNA (100 ng/ml) or control siRNA (100 ng/ml) for 24 hours. The cell viability was measured by MTT reduction activity. rS100A9 protein did not attenuate the Aβ1-42-induced cytotoxicity. rS100A9 alone in the absence of Aβ1-42 also had little effect on the cell viability. Extracellular depletion of S100A9 with siRNA did not induce the cytotoxicity. Values are expressed as the means ± SEM of triplicate experiments. MTT, 3(4,5-dimethylthiazol-2-yl)-2,5-diphenyltetrazolium bromide.

To further describe the pathological mechanism related to S100A9 in AD, the role of extracellular S100A9 depletion related to the Aβ1-42-induced cytotoxicity was investigated. As shown in Figure 5, Aβ1-42 treatment significantly increased cytotoxicity as measured by MTT reduction assay. Addition of rS100A9 protein into the cell culture supernatant did not significantly attenuate the Aβ1-42-induced cytotoxicity (Figure 5A). Treatment with rS100A9 alone in the absence of Aβ1-42 at concentrations up to 10 μg/ml had little effect on the cell viability and, as expected, a similar effect was observed with rS100A9_hi_ (Figure 5B). In addition, depletion of S100A9 with siRNA did not significantly evoke cell toxicity (Figure 5C). These results demonstrate that extracellular depletion of S100A9 was not directly associated with the cytotoxicity in response to mostly Aβ1-42 monomers in human monocytic THP-1 cells.

### Aβ1-42-induced extracellular S100A9 depletion resulted in decreased antimicrobial activity

**Figure 6:**
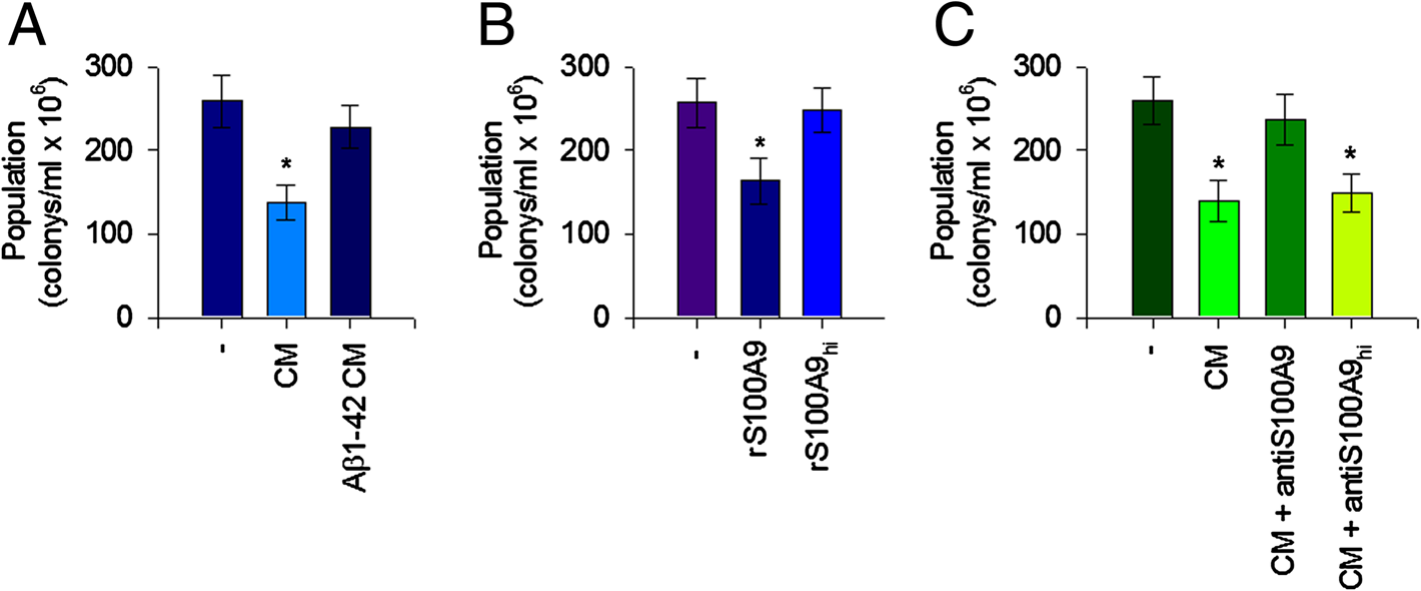
**Aβ1-42-induced extracellular S100A9 depletion resulted in decreased antimicrobial peptide activity.** The antimicrobial activities against *E coli* were assessed with the supernatants from vehicle or Aβ-42 treated THP-1 cells. (**A**) *E coli* were cultured with serum-free RPMI-1640 media alone or the conditioned media from THP-1 cells treated with the vehicle or Aβ1-42 (10 μM) for 24 hours. (**B**) To measure the antimicrobial activities of rS100A9 protein, *E coli* was cultured with rS100A9 or hi-rS100A9 (each 10 μg/ml). (**C**) To inactivate antimicrobial activity of S100A9, which was released into the supernatants, the conditioned media from THP-1 cells treated with the vehicle for 24 hours were pretreated with anti-S100A9 antibodies or heat inactivated anti-S100A9 antibodies for 2 hours at 37°C. *E coli* were then cultured with serum-free RPMI-1640 media alone or the conditioned media treated with anti-S100A9 antibodies or heat inactivated anti-S100A9 antibodies (anti-S100A9_hi_) as indicated. Data showed that vehicle treated supernatant, which contained a significant amount of S100A9, demonstrated antimicrobial activity against *E coli*. Moreover, rS100A9 protein clearly elicited the antimicrobial peptide activity *in vitro.* Immunodepletion of S100A9 blocked antimicrobial activity of the vehicle treated supernatant. All data are presented as the means ± SEM (n = 3). **P <0.01, versus vehicle treated samples. CM, conditioned media.

Recent reports suggested that S100A9 acts as an additional antimicrobial peptide in the innate immune system, which provides immediate protection for the host against microbial challenge by recognizing the presence of microorganisms and preventing their tissue invasion, thus limiting microbial proliferation and inflammation [11,12]. We further investigated the antimicrobial activity of S100A9, which was released into the cell culture supernatants of THP-1 monocytes. The antimicrobial activities against *E coli* were assessed with the cell culture supernatants from vehicle or Aβ-42 treated THP-1 cells. Vehicle treated supernatant, which contained a significant amount of S100A9, demonstrated antimicrobial activity against *E coli*. However, microbial growth was not decreased by the supernatant from Aβ1-42 treated THP-1 cells in which the S100A9 level was significantly reduced (Figure 6A). Moreover, rS100A9 protein clearly elicited the antimicrobial peptide activity *in vitro* (Figure 6B), whereas rS100A9_hi_ had little activity. Consistently, immunodepletion of S100A9 with anti-S100A9 antibodies blocked antimicrobial activity of the vehicle treated supernatant (Figure 6C), confirming that the antimicrobial activity in the vehicle treated supernatant is S100A9-specific.

## Discussion

The present study has four main findings concerning a mechanistic link between S100A9 and AD pathology. First, the mostly monomeric form of Aβ1-42 markedly decreased S100A9 release into the cell culture supernatant of human THP-1 monocytes in parallel with increased intracellular S100A9. Second, this reduction of S100A9 release was accompanied by increased intracellular Ca^2+^ level. Third, depletion of extracellular S100A9 in response to Aβ1-42 monomers was not associated with Aβ1-42-induced cytotoxicity. Finally, Aβ1-42-induced extracellular S100A9 depletion decreased antimicrobial activity of the culture supernatant from human monocytes, which was pathogenically challenged with Aβ1-42. Our findings suggest that mostly Aβ1-42 monomers negatively regulates the innate immune system by down-regulating the secretion of S100A9, which is likely a main mediator of the antimicrobial activity in the culture supernatants of human THP-1 monocytes.

S100A8, S100A9 and S100A12, as endogenous proteins associated with inflammation, are proposed to act as damage-associated molecular pattern (DAMP) initiators of innate immunity [28]. They are found at high concentrations in inflamed tissue, where neutrophils and monocytes are the most abundant cell types, and are released following neutrophil necrosis [29]. S100A8/S100A9 secretion may occur during interaction of phagocytes with endothelial cells and/or stimulation by lipopolysaccharide; IL-1β and TNF can promote S100A8/S100A9 release from monocytes [30,31]. Secretion may involve an energy-dependent process requiring protein kinase C activation in combination with a second calcium-dependent signal and interactions with microtubules [31,32]. Consistent with previous results that activated murine macrophages and human monocytes secreted significant amounts of S100A8 [33,34], this study has shown that human THP-1 monocytes secreted significant amounts of S100A9, which might be involved in autocrine/paracrine activities underlining the inflammatory process; although underlying molecular mechanisms of S100A9 secretion in human THP-1 monocytes remains to be determined.

S100A9 was increased within neuritic plaques and reactive glia, and was proposed to participate in the neuroinflammation associated with the pathogenesis of AD [17]. A recent study also reported that S100A9 interacts with Aβ1-40 and induces its fibrillization, further supporting its association with AD [20]. Consistent with previous observations, our recent study has shown that S100A9 expression was increased in the brains of Tg2576 mice and AD patients [18]. The toxic oligomeric forms of Aβ increased intracellular S100A9 levels in parallel with increases of [Ca^2+^]_i_ and up-regulated S100A9 was found to be involved in the production of proinflammatory cytokines in BV2 cells [18]. Together, these findings propose the potential role of excessive S100A9 expression elicited by Aβ oligomers in the neuroinflammation related to the learning and memory impairment in AD patients, and suggest S100A9 as a possible target for the pathogenesis of AD [18,19]. On the other hand, it is noteworthy that the present study has shown for the first time, to our knowledge, that the mostly monomeric form of Aβ1-42 led to a marked decrease of S100A9 secretion, accompanied by a mild increase of intracellular Ca^2+^ level in human THP-1 monocytes. Furthermore, since S100A9 has Ca^2+^ binding capacity, this extracellular depletion of S100A9 in response to Aβ1-42 monomers appears to be a consequence of increased intracellular S100A9 in parallel with the increased [Ca^2+^]_i_. A recent study has demonstrated a link between extracellular Ca^2+^ entry and a formation of Ca^2+^-dependent heterocomplexes of S100A9, which is a probable prerequisite for its intracellular biological activities such as nicotinamide adenine dinucleotide phosphate-oxidase (NADPH oxidase) activation in myeloid cells [35]. This association of increased Ca^2+^ level with increased intracellular heterotetramers of S100A9 strongly supports our study.

The oligomeric forms of Aβ exhibit stronger cytotoxicity than the monomeric form or the less toxic insoluble fibrillary form through their ability to bind lipid bilayers and cause uncontrolled influx of extracellular Ca^2+^, with devastating consequences for cellular Ca^2+^ homeostasis [36-38]. The present study, in which mostly Aβ1-42 monomers instead of oligomers were used, has demonstrated that Aβ1-42 monomers as measured by MTT assay exhibited cell toxicity in human THP-1 cells. Importantly, depletion of extracellular S100A9 release by Aβ1-42 monomers or siRNA was found to have little effect on the cell viability of human monocytic cells. Moreover, the recombinant S100A9 did not significantly evoke cell toxicity and had little effect on Aβ1-42-induced cytotoxicity in human THP-1 monocytes.

While some aspects of excessive S100A9 could drive disease progression through the inflammation-induced up-regulation of proinflammatory cytokines, as shown in our previous study [18], there is also evidence that S100A9 may exert neuroprotective action. According to published reports, the proinflammatory functions of S100A9 tended to underplay important regulatory, antioxidant and protective properties [9,10]. S100A9 interaction with Aβ1-40 resulted in reduced S100A9 cytotoxicity by the binding of S100A9 toxic species to Aβ1-40 amyloid structures [20]. Consequently, it was implied that secreted S100A9 during inflammation promoted the formation of amyloid plaques and that plaque formation may be the result of a protective response within the brain of AD patients, in part mediated by S100A9 [20]. Taken together, these findings suggest that S100A9 could mediate proinflammatory and anti-inflammatory effects, depending on the monomeric or oligomeric forms of Aβ species [39], the precise protocol used, including the excess or depleted concentrations, duration of exposure, overall immune environment, different cell types and species studied, and disease states; although the reason why S100A9 apparently mediated different effects on cell toxicity is not yet understood. Further studies are needed to clarify the apparent controversy, and to determine both intracellular and extracellular S100A9 using the toxic oligomeric form of Aβ1-42.

Antimicrobial peptides may serve as a line of defense, and defensins are a family of antimicrobial peptides [40]. A previous report suggested that S100A9 (MRP14) is an additional antimicrobial peptide that forms calprotectin (MRP8/14) heterodimer with S100A8 (MRP8) [41]. Consequently, acting as an antimicrobial peptide in the innate immune system, S100A9 could provide immediate protection for the host against microbial challenge by recognizing the presence of microorganisms and preventing their tissue invasion, thus limiting microbial proliferation and inflammation. It is noteworthy that S100A9 is released more from damaged cells and may play a major antimicrobial role [42]. Importantly, our results have shown that Aβ1-42-induced extracellular S100A9 depletion resulted in decreased antimicrobial activity of the culture supernatant of human THP-1 monocytes. This observation was confirmed by immunodepletion of S100A9 with anti-S100A9, which decreased the antimicrobial activity of the culture supernatant of the vehicle treated cells. Furthermore, the recombinant S100A9 elicited the antimicrobial peptide activity *in vitro*.

This is the first report to demonstrate that the mostly monomeric soluble form of Aβ1-42 negatively regulates the innate immune system by down-regulating the secretion of S100A9, which subsequently reduces the S100A9-dependent antimicrobial peptide activity in the culture supernatants of human THP-1 monocytes. This finding stands in stark contrast to recent reports demonstrating that Aβ1-42 possess antimicrobial activity to kill bacteria under the appropriate conditions, which favor the formation of oligomers of Aβ peptide [25,43]. Further research will be required to demonstrate whether the oligomeric form of Aβ1-42 would act together or in parallel with S100A9 to exert its antimicrobial property, and how different forms of Aβ species such as the toxic oligomeric form of Aβ1-42 versus the less toxic monomeric form of Aβ1-42 dysregulate or play a host defense role *in vivo*.

A large body of data supports a central role for neuroinflammation in AD neuropathology and Aβ as the source of AD-associated inflammation [4,6]. Given that inflammatory response in the immunologically privileged CNS is mediated by the innate immune system, our data raise the possibility that rather than Aβ acting as a sole independent initiator of neuroinflammation, increased Aβ may trigger dysregulation of the innate immune system through depletion of extracellular S100A9 release from monocytes and decrease of its antimicrobial activity to protect against invading microbes. Increased microbial infection may further trigger a self-perpetuating innate immune response leading to an inappropriate inflammatory response in the CNS and subsequent production of Aβ, although the underlying cause of the aberrant neuroinflammation in AD patients still remains unclear. A number of studies reporting infection of the CNS of AD patients with various microbial pathogens [44-48] strongly support our study.

## Conclusion

Collectively, our data indicate that Aβ1-42 monomers decrease the secretion of S100A9 in situations where Aβ1-42 enhances cytotoxicity. Furthermore, our findings suggest that the mostly monomeric form of Aβ1-42 negatively regulates the innate immune system by down-regulating the extracellular release of S100A9, which possesses antimicrobial peptide activity in human monocytes. The results of this study, at least in part, support the notion that increased amounts of Aβ1-42 are not only toxic to human monocyte but also disrupt its normal physiological role for a host defense in the innate immune system, thereby contributing to an increased microbial infection in AD patients. Consequently, the results of this study have important implications for ongoing and future AD treatment strategies. However, the relevance of these findings *in vivo* remains to be clearly elucidated.

## Abbreviations

ActD: Actinomycin; AD: Alzheimer’s disease; ANOVA: Analysis of variance; Aβ: Amyloid beta; BAPTA: 1,2-bis(o-aminophenoxy)ethane-N,N,N',N'-tetraacetic acid; CFU: Colony forming unit; CHX: Cycloheximide; CNS: Central nervous system; DAMP: Damage-associated molecular pattern; EGTA: Ethylene glycol tetraacetic acid; MTT: 3(4,5-dimethylthiazol-2-yl)-2,5-diphenyltetrazolium bromide; NADPH oxidase: Nicotinamide adenine dinucleotide phosphate-oxidase; PBMC: Peripheral blood mononuclear cells; rS100A9: Recombinant S100A9; rS100A9hi: Heat-inactivated rS100A9; SDS-PAGE: Sodium dodecyl sulfate polyacrylamide gel electrophoresis; siRNA: Small interfering RNA.

## Competing interests

The authors declare that they have no competing interests.

## Authors’ contributions

The work presented here was carried out in collaboration between all authors. EL and JY carried out most of the laboratory experiments, analyzed the data and interpreted the results. KC helped in preparation of the manuscript. YS and YC conceived the idea for the study, and helped in designing methods and experiments. YC critically supervised the complete study. All the authors read and approved the final revised manuscript.
